# Association of biofilm formation with multidrug resistance in burn wound patients isolates of *Pseudomonas aeruginosa* in Sana’a City, Yemen

**DOI:** 10.3389/fmed.2026.1762684

**Published:** 2026-04-15

**Authors:** Ahlam Ali Maher, Abdullah H. Maad, Ahmed Y. Al-Jaufy, Hude Z. Al-Shami, Abdulhakim M. Al-Saban, Abdulrahman Y. Al-Haifi, Yaser Mohammed Al-Worafi, Ansam M. Al-Barq, Eshtiaq A. Al-Yosffi, Hammood Mohammed Naji Saad, Abdullah Ahmed Al-Dhabali, Zaid Abdo Thawaba, Ali Salman Al-Shami

**Affiliations:** 1 Department of Medical Microbiology and Clinical Immunology, Faculty of Medicine and Health Sciences, Sana’a University, Sana’a, Yemen; 2 Department of Pharmaceutics, College of Pharmacy, University of Al-Ameed, Karbala, Iraq; 3 Department of Pharmacy, Jiblah University for Medical and Health Science, Jiblah, Yemen; 4 Department of Microbiology, Faculty of Medicine, Thamar University, Thamar City, Yemen; 5 College of Medical Sciences, Azal University for Human Development, Sana’a, Yemen; 6 Department of Pediatrics, Althawra Hospital, Al Hudaydah, Yemen; 7 Faculty of Pharmacy, Sana’a University, Sana’a, Yemen; 8 School of Pharmacy & Medical Sciences, Lebanese International University, Sana’a, Yemen; 9 Department of Clinical Pharmacy, College of Medicine and Health Sciences, Al-Saeedah University, Sana’a, Yemen

**Keywords:** AlgD, biofilm, burn wound infection, multidrug resistance, pelF, *Pseudomonas aeruginosa*, pslD, Yemen

## Abstract

**Background:**

Burn wound infections (BWIs) represent a major cause of morbidity and mortality among hospitalized burn patients. *Pseudomonas aeruginosa* is recognized for its ability to acquire multidrug resistance (MDR) and form biofilms that enhance virulence and antimicrobial tolerance.

**Objective:**

This study investigated the association between biofilm formation and multidrug resistance among *P. aeruginosa* isolates from burn wound infections in Sana’a City, Yemen.

**Methods:**

A cross-sectional study was conducted at Republic Hospital, Sana’a City, Yemen, from October 2023 to December 2024. A total of 424 burn wound samples were collected and processed using standard microbiological techniques. Antimicrobial susceptibility testing was performed using the Kirby–Bauer disk diffusion method according to Clinical and Laboratory Standards Institute (CLSI) guidelines (CLSI, 2022). Biofilm production was assessed by the microtiter plate method, and polymerase chain reaction (PCR) was used to detect biofilm-associated genes (algD, pslD, and pelF). Statistical analyses were performed using Statistical Package for the Social Sciences (SPSS) version 26.0, with *p* ≤ 0.05 considered significant.

**Results:**

Of 424 burn wound samples, *P. aeruginosa* was the predominant isolate (39.6%), followed by *Klebsiella pneumoniae* (27.1%) and *Staphylococcus aureus* (19.3%). Significant risk factors associated with bacterial isolation included prior antibiotic use (*χ*^2^ = 16.4, *p* = 0.001), wound debridement (*χ*^2^ = 21.6, p = 0.001), and surgical skin grafting (*χ*^2^ = 11.7, p = 0.001). *P. aeruginosa* showed high resistance to ceftazidime (89.8%), cefepime (90.4%), ticarcillin (92.2%), and meropenem (61.9%), while remaining largely sensitive to colistin (97%). Among isolates, 13% were MDR, 21% extensively drug-resistant (XDR), and 51% pan-drug-resistant (PDR) strains. Biofilm formation was observed in 66.4% of *P. aeruginosa* isolates—19.7% strong, 47.0% moderate, and 33.3% non-producers. The biofilm-associated genes algD, pslD, and pelF were detected in 38, 35, and 27% of isolates, respectively. A significant association was observed between strong biofilm formation and the presence of the pslD gene (*χ*^2^ = 4.8, *p* = 0.03), but not with MDR status (*p* > 0.05).

**Conclusion:**

*P. aeruginosa* remains the leading cause of burn wound infections in Sana’a City, Yemen, exhibiting alarmingly high levels of carbapenem and cephalosporin resistance. Although biofilm formation was common, no significant association was found between biofilm production and multidrug resistance. The high prevalence of PDR strains underscores the urgent need for antimicrobial stewardship, routine susceptibility testing, and infection control measures in burn centers.

## Introduction

Burn injuries remain a major global public health concern and are among the leading causes of morbidity and mortality worldwide, particularly in low- and middle-income countries. According to the World Health Organization, approximately 180,000 deaths annually are attributed to burn injuries, with the highest burden occurring in resource-limited regions (37). Burn wound infections (BWIs) represent one of the most serious complications of thermal injuries, frequently leading to sepsis, prolonged hospitalization, increased healthcare costs, and high mortality rates (32, 35). The disruption of the skin barrier, combined with immunosuppression and prolonged hospital exposure, creates an ideal environment for microbial colonization and infection. Both Gram-positive and Gram-negative bacteria are implicated in BWIs; however, Gram-negative pathogens have become increasingly predominant due to their remarkable ability to acquire multidrug resistance (MDR) and persist in hospital environments (1, 38). Among these pathogens, *Pseudomonas aeruginosa* is recognized as one of the majority of the clinically significant opportunistic organisms associated with burn wound infections and healthcare-associated infections (HAIs) globally (2, 40). This organism possesses intrinsic resistance to multiple antimicrobial agents and has a remarkable capacity to acquire additional resistance mechanisms, including *β*-lactamase production, efflux pump overexpression, reduced outer membrane permeability, and genetic mutations (3, 4). These adaptive mechanisms contribute to the emergence of multidrug-resistant (MDR), extensively drug-resistant (XDR), and pan-drug-resistant (PDR) strains, which significantly complicate treatment and increase mortality risk among burn patients (5, 6, 29). One of the most critical virulence factors contributing to the persistence and antimicrobial resistance of *P. aeruginosa* is its ability to form biofilms (28). Biofilms are structured microbial communities embedded within a self-produced extracellular polymeric matrix composed of exopolysaccharides, extracellular DNA, and proteins (7, 35). This matrix serves as a physical and biochemical barrier that protects bacterial cells from antimicrobial agents and host immune responses. Studies have demonstrated that bacteria within biofilms may exhibit up to 1,000-fold greater resistance to antibiotics compared with planktonic cells (8). This increased tolerance is attributed to multiple mechanisms, including restricted antibiotic penetration, reduced metabolic activity of sessile cells, activation of stress response pathways, and enhanced horizontal gene transfer within biofilm communities (3, 9). Recent studies have increasingly emphasized the strong association between biofilm formation and antimicrobial resistance in *P. aeruginosa*. A meta-analysis conducted by (10) reported that biofilm-producing isolates exhibited significantly higher resistance rates across multiple antibiotic classes than non-biofilm-producing strains. Similarly, biofilm formation has been associated with increased expression of resistance genes, efflux pump systems, and protective stress-response mechanisms, all of which contribute to bacterial survival under antimicrobial pressure (9, 11). Moreover, biofilms facilitate genetic exchange between bacterial cells, enabling the rapid dissemination of resistance determinants and further promoting the emergence of highly resistant strains (12). Biofilm development in *P. aeruginosa* is regulated by several key genes involved in exopolysaccharide production, including algD, pslD, and pelF. The algD gene is essential for alginate synthesis, which contributes to biofilm structural integrity and immune evasion. The pslD gene plays a critical role in bacterial adhesion and biofilm initiation, while the pelF gene contributes to the formation of Pel polysaccharide, which enhances biofilm stability and antimicrobial tolerance (13–15). These biofilm-associated genes are considered key determinants of bacterial persistence and chronic infection (31). The clinical impact of biofilm-associated infections is particularly significant in burn patients, where biofilm formation contributes to chronic infection, delayed wound healing, and increased resistance to antimicrobial therapy (16, 17). Despite substantial global evidence linking biofilm formation to antimicrobial resistance, there is a significant data gap from resource-limited settings, including Yemen. Investigating the relationship between biofilm formation, biofilm-associated genes (algD, pslD, and pelF), and antimicrobial resistance in *P. aeruginosa* clinical isolates is crucial for enhancing treatment strategies, informing antimicrobial stewardship, and reinforcing infection control measures. This study aims to examine the association between biofilm formation, biofilm-associated genes, and multidrug resistance in *P. aeruginosa* isolates obtained from burn wound infections in Sana’a City, Yemen. To our knowledge, this is the first study of its kind in Yemen, providing novel insights into the prevalence of pan-drug-resistant (PDR) strains in a region where data on antimicrobial resistance and biofilm-related virulence factors remain scarce.

## Methodology

### Study design and setting

This cross-sectional study was conducted at the Burn Unit of Republic Hospital, Sana’a City, Yemen, between October 2023 and December 2024. Laboratory analyses were performed at the Department of Microbiology, Republic Hospital, and the Molecular Biology Laboratory, Sana’a University.

### Study population and sample size

A total of 424 burn wound patients were enrolled, including 269 men and 155 women, aged ≤1 to 80 years (mean age: 23.6 years). The sample size was determined using Epi Info version 6 based on a 95% confidence interval (CI) level and a previous local prevalence of *P. aeruginosa* in burn wounds of 49% (43). Inclusion criteria comprised patients hospitalized for >48 h with clinical evidence of burn wound infection. Patients with repeated isolates or hospitalized <48 h were excluded.

### Data collection and clinical variables

Demographic and clinical data were collected through standardized questionnaires, including age, sex, burn type and degree, antibiotic history, comorbidities, surgical interventions, and hospitalization duration. Risk factors associated with bacterial isolation and resistance were analyzed.

#### Inclusion criteria

- Patients who were clinically presumptive for various HAIs were included in the study.
- Patients who had been admitted for more than 48 h and had clinical evidence of hospital-acquired infections at any age and both sexes.

#### Exclusion criteria

- Patients with repeated isolates.

### Specimens’ collection

Wound or pus samples were obtained following Levine’s techniques. Purulent exudates, pus, and discharges were collected aseptically from the depth of the incision with a syringe or sterile cotton swabs dipped in normal saline. The cotton swab was inserted into a tube of Brain Heart Infusion transport medium, transferred within 30 min, and kept at −20 °C (43).

### Isolation and identification of organisms

Blood and MacConkey agar were used to culture the swab specimen, which was incubated aerobically at 35 °C–37 °C for 18–24 h. Following aerobic incubation, Gram staining was used to assess the morphology of the bacteria, followed by routine biochemical assays, such as pigment production in agar, oxidase, urease, catalase, and growth on SIM medium (sulfide, indole, and motility) as well as triple sugar iron agar (TSI) to determine the bacterial species (18). Antibiotic susceptibility was determined for the selected antibiotics by the disk diffusion method according to the Clinical and Laboratory Standards Institute (CLSI) recommendations (36).

### Antimicrobial susceptibility testing and resistance classification

The antimicrobial resistance profiles of *P. aeruginosa* isolates were classified according to the definitions provided by Magiorakos et al. (19):

Multidrug-resistant (MDR) strains: Strains resistant to at least one antibiotic in three or more antimicrobial categories.
Extensively drug-resistant (XDR) strains: Strains resistant to at least one agent in all but two or fewer antimicrobial categories.
Pan-drug-resistant (PDR) strains: Strains resistant to all agents in all antimicrobial categories.

These classifications were used to categorize isolates based on their resistance profiles determined by the Kirby–Bauer disk diffusion method.

### Antimicrobial susceptibility testing

Antibiotic susceptibility was determined by the Kirby–Bauer disk diffusion method on Mueller–Hinton Agar, following the Clinical and Laboratory Standards Institute guidelines (36). A 0.5 McFarland bacterial suspension of *P. aeruginosa* was used for inoculation. The antibiotic disks tested included the following:

- *β*-Lactams: Piperacillin (100 μg), ticarcillin (75 μg), carbenicillin (100 μg), and piperacillin–tazobactam (100/10 μg).
- Cephalosporins: Ceftazidime (30 μg), cefepime (30 μg), and cefoperazone (75 μg).
- Carbapenems: Imipenem (10 μg), meropenem (10 μg), and cefiderocol (30 μg).
- Aminoglycosides: Amikacin (30 μg), gentamicin (10 μg), tobramycin (10 μg), and netilmicin (30 μg).
- Fluoroquinolones: Ciprofloxacin (5 μg), levofloxacin (5 μg), ofloxacin (5 μg), lomefloxacin (5 μg), and gatifloxacin (5 μg).
- Polymyxins: Colistin (10 μg).

### Biofilm detection

Biofilm formation was assessed using the Tissue Culture Plate (TCP) method as previously described by Wu et al. (20) and Sayal et al. (21). First, organisms isolated from fresh agar plates were inoculated in 10 mL of nutrient broth with 1% glucose. Broths were incubated at 37 °C for 24 h. Second, the cultures were then diluted 1:100 with fresh medium. Then, individual wells of sterile 96-well flat-bottom polystyrene tissue culture-treated plates were filled with 200 μL of the diluted cultures, and the negative control well was inoculated with sterile nutrient broth without the organism. The plates were then incubated at 37 °C for 24 h. After incubation, the contents of each well were removed by gentle tapping. After that, the wells were washed 4 times with 0.2 mL phosphate-buffered saline (pH 7.2) to remove free-floating bacteria. Then biofilms formed by bacteria adherent to the wells were fixed by 2% sodium acetate and stained with crystal violet (0.1%), and excess stain was removed by using deionized water, and the plates were kept for drying. Finally, the optical density (OD) of the stained adherent biofilm was determined using a micro-Enzyme-Linked Immunosorbent Assay (micro-ELISA) auto reader at 570 nm and was considered an index of bacteria adhering to the surface and forming biofilms. All the samples were tested in triplicate and repeated 3 times. To validate the biofilm formation assay, we included known control strains:

**Biofilm-positive control:** *P. aeruginosa* PAO1, a well-characterized biofilm-forming strain.

**Biofilm-negative control:** *P. aeruginosa* PA14, a strain known to exhibit weak or no biofilm formation under the conditions used in this study.

### Statistical analysis

Data were analyzed using SPSS version 26.0 and Epi Info version 6.0. Descriptive statistics were used to summarize demographic and microbiological data. Associations between categorical variables were determined using Pearson’s chi-square or Fisher’s exact test, as appropriate. Odds ratios (ORs) with 95% confidence intervals (CIs) were calculated. Statistical significance was set at *p* ≤ 0.05.

## Results

In total, 424 bacterial isolates were obtained from burn wound patients, of which *P. aeruginosa* accounted for 168 (39.6%). The majority of the *P. aeruginosa* isolates were recovered from patients aged >36 years (24.4%), followed by those aged 26–35 years (21.4%) and 16–25 years (20.8%). The majority were men (63.1%), urban residents (56%), and university-educated (44.6%). Children not working (42.9%) and employees (37.5%) represented the main occupational groups, while 55.9% were married. Among patients infected with other bacterial species (256; 60.4%), the majority were also aged >36 years (23.8%), men (63.7%), and urban residents (54.7%). University and secondary education levels predominated (35.5 and 35.2%, respectively), with children (50.4%) and employees (36.3%) forming the largest occupational categories. Married patients constituted 54.7% of this group, as shown in Table 1.

**Table 1:** Distribution of bacterial isolates according to different sociodemographic characteristics among burn wound patients in Sana’a City.

Sociodemographic characteristics		Bacterial types (n = 424)			
		*Pseudomonas aeruginosa* *N* = 168		Others bacterial *N* = 256	
		Number	%	Number	%
Age	0–2	15	8.9	29	11.3
	3–10	24	14.3	51	19.9
	11–15	17	10.1	26	10.2
	16–25	35	20.8	53	20.7
	26–35	36	21.4	36	14.1
	>36	41	24.4	61	23.8
Gender	Male	106	63.1	163	63.7
	Female	62	36.9	93	36.3
Residence	Rural	74	44	116	45
	Urban	94	56	140	55
Educational	Illiterate	31	18.5	56	21.9
	Primary	12	7.1	19	7.4
	Secondary	50	29.8	90	35.2
	University	75	44.6	91	35.5
Occupation	Not working	72	42.9	129	50.4
	Farmer	0	0	3	1.2
	Housewife	33	19.6	31	12.1
	Employee	63	37.5	93	36.3
Marital status	Single	74	44	140	55
	Married	94	56	116	45

Table 2 shows a statistically significant relationship between bacterial type and occupation (*χ*^2^ = 7.1, *p* = 0.07), marital status (*χ*^2^ = 4.5, *p* = 0.03), Qat chewing (*χ*^2^ = 4.3, *p* = 0.03), prior antibiotic use (*χ*^2^ = 16.4, *p* = 0.001), burn wound debridement (*χ*^2^ = 21.6, *p* = 0.001), and surgical skin transplantation (*χ^2^* = 11.7, *p* = 0.001). However, no significant association was found with mechanical ventilation, urinary catheterization, intravenous fluid administration, or central line use (*p* > 0.05). These findings suggest that prior antibiotic exposure and invasive wound management procedures were key risk factors for bacterial infection in burn patients.

**Table 2:** Risk factors and clinical interference among bacterial isolates in burn wound patients, Sana’a City.

Risk factors and clinical interfering		Bacterial isolate				Total *N* = 424		OR	95% CI		*χ^2^*	*p*
		*Pseudomonas aeruginosa* *N* = 168		Others bacterial *N* = 256					L	U		
		Number	%	Number	%	Number	%					
Occupation	Not working	70	42.9	129	50.4	201	47.4				**7.1**	**0.07**
	Farmer	0	0	03	1.2	03	0.7					
	Housewife	33	19.6	31	12.1	64	15.1					
	Employee	63	37.5	93	36.3	156	36.8					
Marital status	Single	74	44	140	55	214	50.4	0.6	0.4	0.9	**4.5**	**0.03**
	Married	94	56	116	45	210	49.6					
Smoking	Yes	52	31	67	26	119	28.0	1.3	0.8	1.9	**1.1**	**0.3**
	No	116	69	189	74	305	71.9					
Qat chewing	Yes	107	64	137	54	244	57.5	1.5	1.02	2.3	**4.3**	**0.03**
	No	61	36	119	46	137	32.3					
Antibiotics uses	Yes	135	80	239	93	374	88	0.3	0.2	0.5	**16.4**	**0.001**
	No	33	20	17	7	50	12					
Mechanical ventilation	Yes	26	15	43	17	69	16	0.9	0.5	1.5	**0.1**	**0.7**
	No	142	85	213	83	355	84					
Urinary catheter	Yes	23	14	34	13	57	13	1.1	0.6	1.8	**0.01**	**0.9**
	No	145	86	222	87	367	87					
Burn wound debridement	Yes	131	78	239	93	370	87	0.3	0.1	0.5	**21.6**	**0.001**
	No	37	22	17	7	54	13					
Surgical skin transplant	Yes	14	8	53	21	67	16	0.3	0.18	0.7	**11.7**	**0.001**
	No	154	92	203	79	357	84					
Intravenous fluid	Yes	161	96	246	96	407	96	0.9	0.3	2.5	**0.02**	**0.9**
	No	07	4	10	4	17	4					
Central line intravenous	Yes	96	57	168	66	264	62	0.7	0.5	1.04	**3.1**	**0.07**
	No	72	43	88	34	160	38					
Medical discharge	Alive	143	85	203	79	346	82	1.5	0.9	2.5	**2.3**	**0.1**
	Dead	25	15	53	21	78	18					

Bold values indicate statistically significant associations (p ≤ 0.05). Odds ratio (OR) ≥ 1 indicates increased risk of infection; *χ*^2^ ≥ 3.83 is significant. *p*-value ≤ 0.05 is significant; CI, confidence interval at 95%. L, means – lower limit; U, means – upper limit.

Table 3 illustrates the relationship between bacterial isolates and clinical outcomes among burn wound patients. No significant association was observed between the type of bacterial isolate and hospital department (*χ^2^* = 1.1, *p* = 0.6). However, a statistically significant association was found between bacterial type and hospitalization duration (*χ^2^* = 28.6, *p* = 0.001), with the majority of infections occurring in patients hospitalized for 3–7 days.

**Table 3:** Clinical outcome and bacterial isolate among burn wound patients in Sana’a City.

Clinical outcome		Bacterial isolates				Total *N* = 424		*χ^2^*	*p*
		*Pseudomonas aeruginosa* *N* = 168		Others bacterial *N* = 256					
		No.	%	No.	%	No.	%		
Hospitalization department	ICU	48	29	69	27	117	28	**1.1**	**0.6**
	Male burn department	67	40	115	45	182	43		
	Female burn department	53	31	72	28	125	29		
Duration of hospitalization	>3	26	15	06	2	32	8	**28.6**	**0.001**
	4–7	110	65	171	67	281	66		
	8–14	25	15	63	25	88	21		
	>15	07	4	16	6	23	5		

*χ*^2^ ≥ 3.83 is (significant); *p*-value ≤ 0.05 is significant. Bold values indicate statistically significant associations between variables (*p* ≤ 0.05).

Table 4 shows that *P. aeruginosa* was the predominant isolate (168; 39.6%), followed by *Klebsiella pneumoniae* (115; 27.1%) and *Staphylococcus aureus* (82; 19.3%). Other isolates included *Citrobacter* spp. (34; 8.0%), *Escherichia coli* (10; 2.4%), and *Streptococcus* spp. (7; 1.7%), while *Acinetobacter* spp. and *Proteus* spp. (3; 0.7% each) and *Enterobacter* spp. (2; 0.5%) were the least frequently detected.

**Table 4:** Frequency of bacterial isolates among burn wound patients in Sana’a City.

Bacterial isolate		
	Frequency	%
*Pseudomonas aeruginosa*	168	39.6
*Klebsiella* spp.	115	27.1
*Staphylococcus aureus*	82	19.3
*Citrobacter* spp.	34	8.0
*Escherichia coli*	10	2.4
*Streptococcus* spp.	7	1.7
*Acinetobacter* spp.	3	0.7
*Proteus* spp.	3	0.7
*Enterobacter* spp.	2	0.5
Total	**424**	**100.0**

Bold values indicate the most prevalent bacterial isolates.

Table 5 illustrates that the highest susceptibility was observed to colistin (163; 97%), followed by imipenem (60; 35.7%) and meropenem (50; 29.8%). In contrast, the highest resistance rates were recorded against carbenicillin (162; 96.4%), piperacillin (158; 94.1%), ticarcillin (155; 92.2%), cefepime (152; 90.4%), and ceftazidime (151; 89.8%). The lowest resistance was noted for colistin (5; 2.9%), imipenem (98; 57.7%), meropenem (104; 61.9%), and netilmicin (120; 71.4%), indicating that colistin remained the most effective agent against *P. aeruginosa* isolates.

**Table 5:** Antibiogram susceptibility pattern of isolated *Pseudomonas aeruginosa* among burn wound patients, Sana’a City.

*Pseudomonas aeruginosa* *N* = 168						
Antibiotics pattern	Susceptible		Intermediate		Resistance	
	Number	%	Number	%	Number	%
Carbenicillin	3	1.7	3	1.7	162	96.4
Piperacillin	8	4.7	2	1.1	158	94.1
Ticarcillin	11	6.5	2	1.1	155	92.2
Cefepime	11	6.5	5	2.9	152	90.4
Ceftazidime	11	6.5	6	3.5	151	89.8
Aztreonam	12	7.1	6	3.6	150	89.3
Lomefloxacin	20	11.9	3	1.7	145	86.3
Ofloxacin	22	13.1	2	1.1	144	85.7
Piperacillin/tazobactam	24	14.3	4	2.4	140	83.3
Amikacin	30	17.9	6	3.5	132	78.6
Ciprofloxacin	31	18.4	5	2.9	132	78.6
Levofloxacin	28	16.7	9	5.4	131	77.9
Tobramycin	34	20.2	9	5.4	125	74.4
Gatifloxacin	35	20.8	9	5.4	124	73.8
Netilmicin	44	26.1	4	2.4	120	71.4
Meropenem	50	29.8	14	8.3	104	61.9
Imipenem	60	35.7	10	5.9	98	57.7
Colistin	163	97.0	0	0	5	2.9

Table 6 shows very high resistance rates to most tested antibiotics among both *P. aeruginosa* and other bacterial isolates. Significant differences were observed for cefepime (*p* = 0.05), ceftazidime (*p* = 0.04), and imipenem (*p* = 0.03), indicating variation in resistance between bacterial groups. However, no significant differences were found for other antibiotics, suggesting widespread multidrug resistance among burn wound pathogens.

**Table 6:** Antibiogram susceptibility of isolated *Pseudomonas aeruginosa* and other bacterial types among patients with burn wounds.

Antibiogram pattern		Bacterial isolates						*χ^2^*	*p*
		*Pseudomonas aeruginosa N* = 168		Other bacterial *N* = 256		Total *N* = 424			
		Number	%	Number	%	Number	%		
Piperacillin	R	158	94	241	94	399	94	**0.5**	**0.8**
	I	02	1	05	3	07	2		
	S	08	5	10	3	18	4		
Cefepime	R	152	90	243	95	395	93	**5.7**	**0.05**
	I	05	3	01	0	06	1		
	S	11	7	12	5	23	6		
Ceftazidime	R	151	90	243	95	394	93	**6.1**	**0.04**
	I	06	4	08	3	14	3		
	S	11	6	05	2	16	4		
Imipenem	R	97	58	177	69	274	65	**6.7**	**0.03**
	I	10	6	07	3	17	4		
	S	60	36	71	28	131	31		
Ticarcillin	R	152	90	218	85	373	88	**4.9**	**0.09**
	I	02	1	05	2	07	2		
	S	11	7	33	13	44	10		
Carbenicillin	R	162	96	242	95	411	97	**2.2**	**0.3**
	I	03	2	03	1	06	1		
	S	03	2	11	4	14	3		
Piperacillin/tazobactam	R	140	84	221	86	361	85	**2.0**	**0.4**
	I	04	2	09	4	13	3		
	S	24	14	26	10	50	12		
Aztreonam	R	150	89	237	93	387	91	**1.7**	**0.4**
	I	06	4	08	3	14	3		
	S	12	7	11	4	23	5		
Ciprofloxacin	R	132	79	213	83	345	81	**3.6**	**0.2**
	I	05	3	02	1	07	2		
	S	31	18	41	16	72	17		

R, resistant; I, intermediate; S, susceptible. *χ*^2^ ≥ 3.83 is significant. *p*-value ≤ 0.05 is significant. Bold values indicate statistically significant differences between bacterial groups (*p* ≤ 0.05).

Table 7 presents that the algD gene was the most prevalent, detected in 53 isolates (38%), followed by pslD in 50 isolates (35%) and pelF in 38 isolates (27%).

**Table 7:** Distribution of biofilm genes (algD, pslD, and pelF) among the isolated biofilm-forming *Pseudomonas aeruginosa*.

Biofilm gene	Frequency (number 336)			
	Yes (No. 141)		No (number 195)	
	Number	%	Number	%
algD	53	**38**	59	**30**
pslD	50	**35**	62	**32**
pelF	38	**27**	74	**38**
Total	**141**	**100**	**195**	**100.0**

Bold values indicate higher prevalence of biofilm-associated genes among isolates.

Table 8 shows the distribution of the algD, pslD, and pelF genes among strong and moderate biofilm-forming *P. aeruginosa* isolates. In strong biofilm producers, the genes algD, pslD, and pelF were detected in 19 (57.7%), 20 (60.6%), and 15 (45.4%) isolates, respectively, while in moderate biofilm producers they were present in 34 (43%), 30 (37.9%), and 23 (29.1%) isolates, respectively. The algD gene showed no significant association with biofilm strength (OR = 1.8, 95% CI: 0.8–4.1; *χ^2^* = 1.9, *p* = 0.2). A significant association was observed between the pslD gene and strong biofilm formation (OR = 2.5, 95% CI: 1.1–5.8; *χ^2^* = 4.8, p = 0.03), whereas the pelF gene showed a non-significant trend toward association (OR = 2.0, 95% CI: 0.9–4.7; *χ^2^* = 2.5, *p* = 0.09).

**Table 8:** Distribution of biofilm genes (algD, pslD, and pelF) among the strong and moderate biofilm-forming *P. aeruginosa.*

Biofilm genes		Biofilm formation				Total *N* = 112		OR	95% CI		*χ^2^*	*p*
		Strong *N* = 33		Moderate *N* = 79								
		Number	%	Number	%	Number	%		L	U		
**AlgD**	Yes	19	57.7	34	43.0	53	**47**	**1.8**	**0.8**	**4.1**	**1.9**	**0.2**
	No	14	42.4	45	56.9	59	**53**					
**PslD**	Yes	20	60.6	30	37.9	50	**45**	**2.5**	**1.1**	**5.8**	**4.8**	**0.03**
	No	13	39.3	49	62.0	62	**55**					
**PelF**	Yes	15	45.4	23	29.1	38	**34**	**2.0**	**0.9**	**4.7**	**2.8**	**0.1**
	No	18	54.5	56	70.9	74	**66**					

Bold values indicate statistically significant associations (*p* ≤ 0.05); OR ≥ 1 indicates increased likelihood of strong biofilm formation; *χ*^2^ ≥ 3.83 is significant. *p*-value ≤ 0.05 is significant; CI, confidence interval at 95%. L, means – lower limit; U, means – upper limit.

Table 9 illustrates the relationship between biofilm-associated genes (algD, pslD, and pelF) and multidrug resistance (MDR) in *P. aeruginosa* isolates. Among MDR isolates, the algD, pslD, and pelF genes were detected in 8 (47.1%), 8 (47.0%), and 4 (23.5%) isolates, respectively. No statistically significant association was observed between the presence of these genes and MDR status (*p* = 0.9, *p* = 0.8, and *p* = 0.3, respectively).

**Table 9:** Biofilm genes (algD, pslD, and pelF) and MDR antibiotic resistance.

Biofilm genes		MDR				Total *N* = 112		OR	95% CI		*χ^2^*	*p*
		Positive *N* = 17		Negative *N* = 95								
		Number	%	Number	%	Number	%		L	U		
**aIgD**	Yes	08	47.1	45	47.3	53	**47**	**0.9**	**0.4**	**2.8**	**0.006**	**0.9**
	No	09	52.9	50	52.7	59	**53**					
**psID**	Yes	08	47.0	42	44.2	50	**45**	**1.1**	**0.4**	**3.2**	**0.04**	**0.8**
	No	09	52.9	53	55.8	62	**55**					
**PelF**	Yes	04	23.5	34	35.8	38	**34**	**0.6**	**0.2**	**1.8**	**0.9**	**0.3**
	No	13	76.5	61	64.2	74	**66**					

OR, odds ratio ≥ 1 is at risk of infection; *χ*^2^ ≥ 3.83 is significant. *p*-value ≤ 0.05 is significant; CI, confidence interval at 95%. L, means – lower limit; U, means – upper limit. Bold values indicate statistically significant associations between biofilm genes and MDR (*p* ≤ 0.05).

Table 10 presents the association between biofilm-related genes (algD, pslD, and pelF) and extensively drug-resistant (XDR) *P. aeruginosa* isolates. The algD, pslD, and pelF genes were detected in 11 (44%), 12 (48%), and 11 (44%) of XDR isolates, respectively. No statistically significant correlation was observed between the presence of these genes and XDR resistance (*p* = 0.7, p = 0.7, and p = 0.2, respectively).

**Table 10:** Biofilm gene (algD, pslD, and pelF) and XDR antibiotic resistance.

Biofilm genes		XDR				Total		OR	95% CI		*χ^2^*	*p*
		Positive *N* = 25		Negative *N* = 87								
		Number	%	Number	%	Number	%		L	U		
**aIgD**	Yes	11	44	42	48	53	**47**	**0.8**	**0.3**	**2.1**	**0.1**	**0.7**
	No	14	56	45	52	59	**53**					
**psID**	Yes	12	48	38	44	50	**45**	**1.2**	**0.5**	**2.9**	**0.1**	**0.7**
	No	13	52	49	56	62	**55**					
**pelF**	Yes	11	44	27	31	38	**34**	**1.7**	**0.7**	**4.3**	**1.5**	**0.2**
	No	14	56	60	69	74	**66**					

OR, odds ratio ≥ 1 is at risk of infection; *χ*^2^ ≥ 3.83 is significant. *p*-value ≤ 0.05 is significant; CI, confidence interval at 95%. L, means – lower limit; U, means – upper limit. Bold values indicate statistically significant associations between biofilm genes and XDR (*p* ≤ 0.05).

Table 11 demonstrates that no significant association was observed between the presence of these genes and PDR status. The algD, pslD, and pelF genes were detected in 23 (43%), 21 (40%), and 14 (26%) of PDR isolates, respectively.

**Table 11:** Biofilm gene (algD, pslD, and pelF) and PDR antibiotic resistance.

Biofilm genes		PDR				Total		OR	95% CI		*χ^2^*	***p***
		Positive *N* = 53		Negative *N* = 59								
		Number	%	Number	%	Number	%		L	U		
aIgD	Yes	23	43	30	51	53	**47**	**0.7**	**0.4**	**1.6**	**0.6**	**0.4**
	No	30	57	29	49	59	**53**					
psID	Yes	21	40	29	49	20	**18**	**0.7**	**0.3**	**1.4**	**1.0**	**0.3**
	No	32	60	30	51	62	**55**					
pelF	Yes	14	26	24	41	38	**34**	**0.5**	**0.2**	**1.2**	**2.5**	**0.1**
	No	39	74	35	59	74	**66**					

OR, odds ratio ≥ 1 is at risk of infection; *χ*^2^ ≥ 3.83 is significant. *p*-value ≤ 0.05 is significant; CI, confidence interval at 95%. L, means – lower limit; U, means – upper limit. Bold values indicate statistically significant associations between biofilm genes and PDR (*p* ≤ 0.05).

## Discussion

In this study, a total of 424 bacterial isolates were recovered from burn wound infections, of which *P. aeruginosa* was the predominant pathogen, accounting for 39.6% of isolates. This finding is consistent with global reports identifying *P. aeruginosa* as one of the most common and clinically significant pathogens in burn wound infections due to its adaptability, intrinsic resistance, and ability to survive in moist hospital environments (2, 34, 40, 41). The predominance of *P. aeruginosa* observed in this study highlights its critical role in burn-associated infections, particularly in healthcare settings where prolonged hospitalization and antibiotic exposure promote colonization and persistence.

The analysis of clinical risk factors demonstrated that prior antibiotic use, wound debridement, and surgical skin grafting were significantly associated with bacterial infection. These findings are supported by previous studies indicating that invasive procedures and antibiotic exposure create selective pressure favoring the emergence and persistence of resistant organisms, particularly in burn units (1, 22). Excessive antibiotic use promotes the selection of resistant strains by eliminating susceptible bacterial populations, allowing resistant organisms such as *P. aeruginosa* to proliferate (6).

The antimicrobial susceptibility profile revealed alarmingly high resistance rates to multiple antibiotic classes, including ceftazidime (89.8%), cefepime (90.4%), and meropenem (61.9%), while colistin remained highly effective (97% susceptibility) (27). These findings are consistent with regional and global reports documenting increasing resistance among *P. aeruginosa*, particularly to *β*-lactams and carbapenems, which are commonly used in clinical practice (5, 23). Carbapenem resistance is of particular concern because these antibiotics are often used as last-line treatments. Resistance mechanisms include carbapenemase production, reduced membrane permeability, efflux pump overexpression, and enzymatic antibiotic degradation (4, 11). The continued effectiveness of colistin observed in this study is consistent with previous reports; however, the increasing global emergence of colistin resistance underscores the importance of cautious and controlled use of this antibiotic (6).

While biofilm formation was common in *P. aeruginosa* isolates, no significant correlation was found between biofilm production and multidrug resistance (MDR), extensively drug-resistant (XDR), or pan-drug-resistant (PDR) phenotypes. This suggests that the relationship between biofilm formation and antimicrobial resistance is more complex than previously assumed. Other resistance mechanisms, such as efflux pump overexpression, reduced membrane permeability, and genetic mutations, may contribute to resistance independently of biofilm formation.

A particularly important and novel finding of this study is the high prevalence of pan-drug-resistant (PDR) strains (51%), in addition to MDR (13%) and XDR (21%) isolates. These rates are higher than those reported in several previous studies conducted in Ethiopia and Iran (5, 18), suggesting a worsening antimicrobial resistance situation in Yemen. The high prevalence of PDR strains represents a significant clinical concern, as these strains are resistant to nearly all available antibiotics, severely limiting treatment options and increasing the risk of treatment failure, prolonged hospitalization, and mortality (39). This finding highlights the urgent need for improved antimicrobial stewardship programs and infection control measures in burn units in Yemen.

Biofilm formation was observed in 66.4% of the *P. aeruginosa* isolates, with 19.7% classified as strong biofilm producers. This prevalence is consistent with previous studies reporting biofilm formation rates ranging from 60 to 80% among clinical isolates of *P. aeruginosa* (7, 24). Biofilm formation is a key virulence factor that contributes significantly to bacterial survival and persistence in clinical environments. The extracellular biofilm matrix acts as a protective barrier that limits antibiotic penetration and protects bacteria from host immune responses (8). In addition, bacteria within biofilms exhibit reduced metabolic activity, which reduces their susceptibility to antibiotics targeting actively dividing cells (3).

Recent molecular studies have demonstrated that biofilms also facilitate horizontal gene transfer, enabling the spread of antibiotic resistance genes within bacterial populations (9). This mechanism contributes to the persistence and dissemination of resistant strains in hospital environments. Therefore, the high prevalence of biofilm formation observed in this study represents an important contributing factor to the persistence and treatment difficulty of *P. aeruginosa* infections (33). The molecular analysis of biofilm-associated genes revealed that the algD, pslD, and pelF genes were present in 38, 35, and 27% of isolates, respectively. These genes play critical roles in biofilm development and structural integrity. The algD gene is essential for alginate biosynthesis, which contributes to biofilm stability and resistance to host immune responses (25). The pslD gene plays a key role in bacterial adhesion and early biofilm formation, while the pelF gene contributes to exopolysaccharide production and biofilm structural stability (14, 15, 30). These findings are consistent with previous studies reporting high prevalence of biofilm-associated genes among clinical *P. aeruginosa* isolates (13, 26). Importantly, a significant association was observed between the presence of the pslD gene and strong biofilm formation. This finding confirms the critical role of the Psl exopolysaccharide in biofilm initiation and structural integrity, as previously reported (15). Psl promotes bacterial adhesion to surfaces, enhances biofilm maturation, and protects bacterial cells from antimicrobial agents and host immune responses (14). However, no statistically significant association was observed between biofilm formation or biofilm-associated genes and MDR, XDR, or PDR phenotypes. This finding suggests that biofilm formation primarily contributes to antimicrobial tolerance rather than to direct genetic resistance. Antibiotic resistance and biofilm formation are complex, multifactorial processes regulated by different genetic and environmental mechanisms (4, 11). Similar findings have been reported in previous meta-analyses, which concluded that while biofilm-forming isolates often exhibit increased resistance, biofilm formation alone is not an independent predictor of multidrug resistance (10).

The novelty of this study lies in its comprehensive analysis of phenotypic biofilm formation combined with molecular detection of key biofilm-associated genes (algD, pslD, and pelF) in clinical isolates of *P. aeruginosa* from burn patients in Yemen. To the best of our knowledge, this is the first study in Yemen to investigate the relationship between biofilm formation, biofilm-associated genes, and multidrug resistance in burn wound isolates of *P. aeruginosa*. Furthermore, the identification of a high prevalence of PDR strains highlights an urgent and previously underreported public health concern in this region.

The coexistence of extensive antimicrobial resistance and biofilm-forming capacity among clinical isolates represents a serious clinical challenge. Biofilm-associated infections are difficult to treat due to their resistance to antimicrobial therapy and immune clearance. Therefore, improved infection control practices, rational antibiotic use, and continuous surveillance of antimicrobial resistance and biofilm-associated virulence factors are essential to prevent the further spread of these highly persistent pathogens.

## Conclusion

This study underscores the alarming prevalence of *P. aeruginosa* as the predominant pathogen responsible for burn wound infections in Sana’a City, Yemen, and its extensive resistance to multiple antibiotic classes. The high frequency of multidrug-resistant (MDR), extensively drug-resistant (XDR), and pan-drug-resistant (PDR) strains presents a significant therapeutic challenge in the management of burn injuries. The detection of biofilm-associated genes (algD, pslD, and pelF) and the strong correlation between pslD expression and robust biofilm formation suggest complex genetic regulation of biofilm development. These molecular findings highlight the importance of integrating gene-level diagnostics with phenotypic testing to enhance clinical management and infection control.

The results emphasize the urgent need to implement antimicrobial stewardship programs, continuously monitor resistance trends, and reinforce infection prevention measures in burn care units. Furthermore, the emergence of PDR *P. aeruginosa* strains underscores the need to explore novel therapeutic approaches, including anti-biofilm agents, bacteriophage therapy, and combination therapies, to combat evolving resistance. Strengthening microbiological capacity in Yemen through molecular surveillance and the adoption of rational antibiotic-use policies is crucial in addressing the escalating challenge of antimicrobial resistance.

## Data Availability

The datasets presented in this study can be found in online repositories. The names of the repository/repositories and accession number(s) can be found in the article/supplementary material.

## References

[1] Gallaher JR Banda W Lachiewicz AM Krysiak R Cairns BA Charles AG. Colonization with MDR Enterobacteriaceae and mortality following burn injury in Sub-Saharan Africa. World J Surg. (2018) 42:3089–96. doi: 10.1007/s00268-018-4633-7, 29696325 PMC6128739

[2] Emami A Pirbonyeh N Keshavarzi A Javanmardi F Moradi Ghermezi S Ghadimi T. Infection profile and antimicrobial resistance pattern from burn patients. Infect Drug Resist. (2020) 13:1499–506. doi: 10.2147/IDR.S249160, 32547119 PMC7246306

[3] Breidenstein EB de la Fuente-Núñez C Hancock RE. *Pseudomonas aeruginosa*: all roads lead to resistance. Trends Microbiol. (2011) 19:419–26. doi: 10.1016/j.tim.2011.04.005, 21664819

[4] Pang Z Raudonis R Glick BR Lin TJ Cheng Z. Antibiotic resistance in *Pseudomonas aeruginosa*: mechanisms and alternative therapeutic strategies. Biotechnol Adv. (2019) 37:177–92. doi: 10.1016/j.biotechadv.2018.11.012 30500353

[5] Addis T Araya S Desta K. Occurrence of multiple, extensive and pan drug-resistant Pseudomonas aeruginosa and carbapenemase production from presumptive isolates stored in a biobank at Ethiopian Public Health Institute. Infect Drug Resist. (2021) 14:3609–18. doi: 10.2147/IDR.S327652, 34511952 PMC8427834

[6] Bassetti M Peghin M Vena A Giacobbe DR. Treatment of infections due to multidrug-resistant gram-negative bacteria. Front Med. (2019) 6:74. doi: 10.3389/fmed.2019.00074, 31041313 PMC6477053

[7] Thi MTT Wibowo D Rehm BHA. *Pseudomonas aeruginosa* biofilms. Int J Mol Sci. (2020) 21:8671. doi: 10.3390/ijms21228671, 33212950 PMC7698413

[8] Høiby N Bjarnsholt T Givskov M Molin S Ciofu O. Antibiotic resistance of bacterial biofilms. Int J Antimicrob Agents. (2010) 35:322–32. doi: 10.1016/j.ijantimicag.2009.12.011, 20149602

[9] Fernández-Billón M Llambías-Cabot AE Jordán-Alluch E Oliver A Macià MD. Mechanisms of antibiotic resistance in biofilms. Biofilm. (2023) 3:100074. doi: 10.1016/j.bioflm.2023.100074, 37205903 PMC10189392

[10] Hadadi-Fishani M Khaledi A Fatemi-Nasab ZS. Correlation between biofilm formation and antibiotic resistance. Infez Med. (2020) 28:47–54. 32172260

[11] Poole K. *Pseudomonas aeruginosa*: resistance to antimicrobials. Front Microbiol. (2011) 2:65. doi: 10.3389/fmicb.2011.00065, 21747788 PMC3128976

[12] Römling U Balsalobre C. Biofilm infections, their resilience to therapy and innovative treatment strategies. J Intern Med. (2012) 272:541–61. doi: 10.1111/j.1365-2796.2012.02559.x, 23025745

[13] Hakime R Salimizand H Khodabandehloo M Fayyazi A Ramazanzadeh R. Prevalence of biofilm genes in *Pseudomonas aeruginosa*. Biomed Res Int. (2022) 2022:1716087. doi: 10.1155/2022/1716087, 35655484 PMC9155974

[14] Huszczynski SM Lam JS Khursigara CM. Role of polysaccharides in biofilm development. Front Microbiol. (2020) 11:1156. doi: 10.3389/fmicb.2020.01156, 32612582 PMC7308725

[15] Whitney JC Colvin KM Marmont LS Robinson H Parsek MR Howell PL. Structure of the Pel polysaccharide. Proc Natl Acad Sci USA. (2015) 112:11353–8. doi: 10.1073/pnas.1503058112, 26311845 PMC4568648

[16] Del Pozo JL Patel R. The challenge of treating biofilm-associated infections. Clin Pharmacol Ther. (2007) 82:204–9. doi: 10.1038/sj.clpt.6100247, 17538551

[17] Pinto RM . Biofilm-related bloodstream infections. Front Microbiol. (2021) 12:681010. doi: 10.3389/fmicb.2021.681010

[18] Dogonchi AA Ghaemi EA Ardebili A Yazdansetad S Pournajaf A. Metallo-β-lactamase-mediated resistance among clinical carbapenem-resistant *Pseudomonas aeruginosa*. Tzu Chi Med J. (2018) 30:90–6. doi: 10.4103/tcmj.tcmj_173_17, 29875589 PMC5968749

[19] Magiorakos AP Srinivasan A Carey RB Carmeli Y Falagas ME Giske CG . Multidrug-resistant, extensively drug-resistant and pandrug-resistant bacteria: an international expert proposal for interim standard definitions for acquired resistance. Clin Microbiol Infect. (2012) 18:268–81. doi: 10.1111/j.14690691.2011.03570.x 21793988

[20] Wu MC Lin TL Hsieh PF Yang HC Wang JT. Isolation of genes involved in biofilm formation of a *Klebsiella pneumoniae* strain causing pyogenic liver abscess. PLoS One. (2011) 6:e23500. doi: 10.1371/journal.pone.0023500, 21858144 PMC3155550

[21] Sayal P Sandhu R Singh K Devi P. Bacterial colonization associated with prolonged catheterization: who is at risk? Int J Res Med Sci. (2017) 5:166–70.

[22] Van Langeveld I Gagnon RC Conrad P Gamelli RL Choudhry MA Mosier MJ. Risk factors for MDR infections in burn centers. Infect Control Hosp Epidemiol. (2017) 38:306–13. doi: 10.1017/ice.2016.266, 27919312

[23] Abdeta A Bitew A Fentaw S Tsige E Assefa D Lejisa T . Phenotypic characterization of carbapenem non-susceptible gram-negative bacilli isolated from clinical specimens. PLoS One. (2021) 16:e0254006. doi: 10.1371/journal.pone.0254006, 34855767 PMC8638961

[24] Rashad FM Abd El-Aziz NK Salem HM . Molecular regulation of biofilm development. Microb Pathog. (2022) 163:105403. doi: 10.1016/j.micpath.2022.105403 35033636

[25] Esoda CN Kuehn MJ. *Pseudomonas aeruginosa* leucine aminopeptidase influences biofilm structure via vesicle-associated antibiofilm activity. MBio. (2019) 10:e02548–19. doi: 10.1128/mBio.02548-19, 31744920 PMC6867898

[26] AL-Mashhadani RKA AboKsour MF Dakhil OAA. Determine Biofilm Genes in Pseudomonas Aeruginosa Isolated from Clinical and Environmental Samples. J. Contemp. Med. Sci. (2024) 10. Available at: https://www.jocms.org/index.php/jcms/article/view/1557.

[27] Alkhudhairy MK Al-Salihy M. Prevalence of metallo-β-lactamase-producing *Pseudomonas aeruginosa* isolated from diabetic foot infections in Iraq. New Microbes New Infect. (2020) 35:100659. doi: 10.1016/j.nmni.2020.100659, 32194966 PMC7076140

[28] Almblad H Rybtke M Hendiani S Andersen JB Givskov M Tolker-Nielsen T. High levels of cAMP inhibit *Pseudomonas aeruginosa* biofilm formation through reduction of c-di-GMP content. Microbiology. (2019) 165:324–33. doi: 10.1099/mic.0.000772 30663958

[29] Altoparlak U Erol S Akcay MN Celebi F Kadanali A. The time-related changes of antimicrobial resistance patterns and predominant bacterial profiles of burn wounds and body flora of burned patients. Burns. (2004) 30:660–4. doi: 10.1016/j.burns.2004.03.005 15475138

[30] Baker P Howell PL Snarr BD Alnabelseya N Pestrak MJ Lee MJ. Exopolysaccharide biosynthetic glycoside hydrolases disrupt *Pseudomonas aeruginosa* biofilms. Sci Adv. (2016) 2:e1501632. doi: 10.1126/sciadv.1501632, 27386527 PMC4928890

[31] Banar M Emaneini M Satarzadeh M Abdellahi N Beigverdi R van Leeuwen WB . Evaluation of enzyme effects on biofilm production of *Pseudomonas aeruginosa* isolated from burn wound infections. PLoS One. (2016) 11:e0164622. doi: 10.1371/journal.pone.0164622, 27736961 PMC5063459

[32] D’Abbondanza JA Shahrokhi S. Burn infection and burn sepsis. Surg Infect. (2021) 22:58–64. doi: 10.1089/sur.2020.086 32364824

[33] Everett J Turner K Cai Q Gordon V Whiteley M Rumbaugh K. Arginine is critical for pathogenesis of *Pseudomonas aeruginosa* in burn wound infections. MBio. (2017) 8:e02160–16. doi: 10.1128/mBio.02160-16, 28292986 PMC5350470

[34] Gupta M Neelam A Singh SK. Bacteriological profile and antimicrobial resistance of burn wound infections in a tertiary care hospital. Heliyon. (2019) 5:e02956. doi: 10.1016/j.heliyon.2019.e02956, 31886427 PMC6921111

[35] Rasamiravaka T Labtani Q Duez P El Jaziri M. The formation of biofilms by *Pseudomonas aeruginosa*. Biomed Res Int. (2015) 2015:759348. doi: 10.1155/2015/759348, 25866808 PMC4383298

[36] Clinical and Laboratory Standards Institute (CLSI). Performance standards for antimicrobial susceptibility testing. 32nd ed. CLSI supplement M100. Wayne (PA): CLSI (2022).

[37] World Health Organization (WHO). Burns. Geneva: WHO (2018). Available at: https://www.who.int/news-room/fact-sheets/detail/burns?utm_source=chatgpt.com

[38] Sheridan RL. Burns in children. J Burn Care Res. (2017) 38:e618–e624. doi: 10.1097/BCR.0000000000000536 28328667

[39] Matos EC Andriolo RB Rodrigues YC Lima PD. Carneiro IC, Lima KV. Mortality of patients with multidrug-resistant infections in intensive care units: a systematic review. Rev Bras Ter Intensiva. (2018) 30:338–45. doi: 10.5935/0103-507X.2018004430328987

[40] Liao C Huang X Wang Q Yao D Lu W. Virulence Factors of Pseudomonas Aeruginosa and Antivirulence Strategies to Combat Its Drug Resistance. Front Cell Infect Microbiol. (2022) 12:926758. doi: 10.3389/fcimb.2022.926758 35873152 PMC9299443

[41] Moradali MF Ghods S Rehm BH. Pseudomonas aeruginosa Lifestyle: A Paradigm for Adaptation, Survival, and Persistence. Front Cell Infect Microbiol. (2017) 7:39. doi: 10.3389/fcimb.2017.00039 28261568 PMC5310132

[42] Murray PR Rosenthal KS Pfaller MA. Medical microbiology. 9th ed. Philadelphia (PA): Elsevier (2020). Available at: https://shop.elsevier.com/books/medical-microbiology/murray/978-0-323-67322-8?utm_source=chatgpt.com

[43] Abdi FA Motumma AN Kalayu AA Abegaz WE. Prevalence and antimicrobial-resistant patterns of Pseudomonas aeruginosa among burn patients attending Yekatit 12 Hospital Medical College in Addis Ababa, Ethiopia. PLoS One. (2024) 19:e0289586. doi: 10.1371/journal.pone.0289586 38452016 PMC10919618

